# Subtropical specialists dominate a coral range expansion front

**DOI:** 10.1007/s00338-024-02601-w

**Published:** 2024-12-16

**Authors:** Fiona Chong, Giun Yee Soong, Agus Alim Hakim, Camille Burke, Stéphane De Palmas, Fabian Gösser, Wanchien Victoria Hsiao, Hiroki Kise, Miyuki Nishijima, Akira Iguchi, Brigitte Sommer, Domino Joyce, Maria Beger, James Davis Reimer

**Affiliations:** 1 Energy and Environment Institute, University of Hull, Hull, UK; 2 Molecular Invertebrate Systematics and Ecology Laboratory, Graduate School of Engineering and Science, University of the Ryukyus, Okinawa, Japan; 3 Geological Survey of Japan, National Institute of Advanced Industrial Science and Technology, Tsukuba, Japan; 4 Department of Aquatic Resources Management, Faculty of Fisheries and Marine Sciences, IPB University, West Java, Indonesia; 5 Department of Earth, Ocean, and Ecological Sciences, University of Liverpool, Liverpool, UK; 6 School of Biology, Faculty of Biological Sciences, University of Leeds, Leeds, UK; 7 Research Laboratory on Environmentally-Conscious Developments and Technologies [E-Code], National Institute of Advanced Industrial Science and Technology, Tsukuba, Ibaraki Japan; 8 School of Life and Environmental Sciences, The University of Sydney, Sydney, NSW Australia; 9 Biological Sciences, University of Hull, Hull, UK; 10 Centre for Biodiversity and Conservation Science, School of the Environment, The University of Queensland, Brisbane, QLD Australia; 11 Tropical Biosphere Research Center, University of the Ryukyus, Okinawa, Japan

**Keywords:** Tropicalisation, *Pocillopora*, Symbiodiniaceae, High latitude, Biogeographic transition zone

## Abstract

Potential range expansion of scleractinian corals in high-latitude reefs is critically dependent on the coral host-symbiont relationship that determines coral growth and survival. Although increases in coral cover have been observed at higher latitudes, the identities of habitat-building reef corals and their symbionts are underreported. Here, we examine how scleractinian host and symbiont Symbiodiniaceae diversity changes along a tropical–temperate environmental gradient. We use *Pocillopora* spp. and associated symbiont communities as a model to understand whether they are expanding their range poleward and the role of symbionts in this process. Along the Kuroshio Current, which carries warm equatorial waters northward along the Pacific coast of Japan, we collected coral tissues from 23 (sub)tropical-to-temperate reefs, from southern Iriomote in the Ryukyu Islands (24°N) to northernmost Kushimoto on mainland Japan (33°N). We examined host identities through direct sequencing of the mitochondrial open reading frame (mtORF) and symbiont identities with next-generation sequencing of the internal transcribed spacer 2 (ITS2) region of the ribosomal DNA. Our results show a dramatic reduction of *Pocillopora* haplotypes and a marked change in dominant symbiont types northward (poleward) from Cape Sata (30°N), Kagoshima. ‘Tropical’ *Pocillopora* haplotypes were absent from mainland Japan sites. We also demonstrate high host specificity between the subtropical *Pocillopora* haplotype and *Cladocopium* symbiont types. Our findings question how common ‘coral tropicalisation’ is, and the location of the coral range expansion front. The specificity of hosts and symbionts in high-latitude corals suggests that high-latitude reefs are unlikely to support the persistence of tropical zooxanthellate corals.

**Supplementary Information:**

The online version contains supplementary material available at 10.1007/s00338-024-02601-w.

## Abstract (zh)

海洋の温暖化に伴った高緯度地域へのサンゴ類の分布拡大は、しばしば「熱帯化」と呼ばれている。イシサンゴ類の成長と生存は、サンゴとその体内に共生する褐虫藻との関係に依存している。日本の高緯度地域において、イシサンゴ類の被度の増加が観察されているが、イシサンゴ類と褐虫類の組成についての報告例は限られている。そこで本研究では、熱帯から温帯の環境勾配に沿って、イシサンゴ類と褐虫類の組成がどのように変化するかを調査した。ハナヤサイサンゴ類と共生する褐虫類をモデル系として、ハナヤサイサンゴ類が分布拡大しているかどうか、またこのプロセスにおける褐虫類の役割を明らかにすることを目的とした。赤道付近の暖かい海水を日本の太平洋岸に沿って流れる黒潮に沿って、琉球列島の西表島南部 (北緯24度) から日本本土の串本最北端 (北緯33度) に至る23地点の熱帯のサンゴ礁から温帯のサンゴ群集でサンゴの断片を採集した。ミトコンドリアのopen reading frame (mtORF) の塩基配列決定によりサンゴの同定を行い、核リボソームDNAのinternal transcribed spacer 2 (ITS2) 領域を対象に次世代シーケンサーを用いて褐虫藻の組成を把握した。その結果、熱帯域特有のハプロタイプを持つハナヤサイサンゴ類は本州では観察されず、鹿児島県佐多岬 (北緯30°) から北へ向かうにつれて、ハナヤサイサンゴ類のハプロタイプが劇的に減少し、共生する褐虫藻の組成も顕著に変化していることが明らかになった。また、亜熱帯域特有のハプロタイプを持つハナヤサイサンゴ類と褐虫藻の*Cladocopium*との間に高い宿主特異性があることも明らかになった。本研究の結果から、高緯度のサンゴ群集におけるイシサンゴ類と褐虫藻の特異性が明らかになり、高緯度のサンゴ群集は熱帯性の有藻性サンゴの存続を支える可能性は低く、高緯度のサンゴ群集が熱帯性の有藻性サンゴの気候的な避難場所として機能する可能性が低いことが示唆された。

## Introduction

Tropicalisation of marine environments is a relatively recent phenomenon arising from anthropogenic climate change (Zarzyczny et al. 2024), where global warming causes species to shift their distributions as suitable environmental conditions shift. For example, subtropical–temperate regions at high latitudes become increasingly more suited for the growth and survival of tropical species (Kumagai et al. 2018). This process predicts a poleward range expansion of some tropical species, accompanied by a reduction in abundance and extent of temperate species (Vergés et al. 2014; Messer et al. 2020). Areas of prolific tropicalisation are typically biogeographic transition zones associated with western boundary currents (Vergés et al. 2014). These currents carry warm, oligotrophic waters from the equator to temperate latitudes, such as the Kuroshio Current in the North Pacific and the East Australian Current in the South Pacific (Imawaki et al. 2013; Sen Gupta et al. 2021), extending suitable conditions for some tropical species beyond the tropics. For example, reefs in New South Wales, Australia, now host more tropical herbivorous fish species than in the early 2000s (Smith et al. 2021). In Japan, *Acropora* coral distribution is estimated to have expanded poleward at ~ 14 km/year since records began in 1930s (Yamano et al. 2011). However, the extent to which ‘tropicalisation by range shift’ occurs in scleractinian coral communities remains a contentious topic, as changes in coral community composition could instead be a result of the increased growth (or proliferation, sensu Keshavmurthy et al. (2023)) of native subtropical/temperate corals that are often cryptic or undescribed (Fifer et al. 2022; Keshavmurthy et al. 2023). This hypothesis is supported by the ongoing discovery and description of subtropical endemic coral species (e.g. coralprojectphoenix.org) with molecular tools (Cowman et al. 2020). In addition, despite having a critical role on the physiology and survival of the coral host (Starko et al. 2023), the identities of symbiotic dinoflagellate Symbiodiniaceae in corals affected by climate change and potential tropicalisation in biogeographic transition zones remains largely unexamined (but see Wicks et al. 2010; Lien et al. 2013).

The tropicalisation of temperate reefs by range expansion is driven by rising ocean temperatures, favouring hard coral growth in previously colder waters, and the deforestation of macroalgal beds by tropical herbivorous fishes (Kumagai et al. 2018; Zarco-Perello et al. 2020), urchins (Ling et al. 2009) or environmental pulses such as storms and heatwaves (Wernberg et al. 2013, 2024). The loss of foundation species, such as kelp at higher latitudes, results in more physical space becoming available for other benthic competitors such as turf algae and hard corals (Vergés et al. 2016; Zarco-Perello et al. 2021). Fossil records from the Holocene, where sea surface temperatures were on average 1.5 °C warmer than today, demonstrated that high-latitude reefs in Tateyama, Japan (~ 35°N) 6000 years ago had twice the number of coral species as compared to coral communities in the vicinity today (Veron and Minchin 1992; Buddemeier et al. 2004). Drawing on this analogue, the observed increase in coral cover at higher latitudes as a result of warming oceans has fueled the debate around the temperate and subtropical reefs acting as climate change refugia for tropical corals (Beger et al. 2014; Muir et al. 2015; de Oliveira Soares 2020), in which range expansion into marginal environments preserves genetic diversity under climate change.

An alternative to ‘tropicalisation by range shift’ explaining the observed increase in coral cover at high latitudes is the proliferation of subtropical species in their existing range (Keshavmurthy et al. 2023). Range expansion is strongly reliant on the corals’ ability to withstand prolonged periods of low winter temperatures (~ 13 °C), which can cause cold bleaching and subsequent mortality (Suzuki et al. 2013; Higuchi et al. 2015), while proliferation of existing subtropical species relies on individuals that must already have this ability. Range expansion by tropical species also relies on a consistent tropical-to-temperate larval supply to ensure population connectivity (Mizerek et al. 2021; Nakamura et al. 2021). A growing body of evidence suggests that high-latitude reefs host more endemic species than previously thought, and these corals could be responsible for the observed ‘subtropical proliferation’. For example, *Pocillopora aliciae*, *Cyphastrea salae* and *Plesiastrea versipora* (Schmidt-Roach et al. 2013b; Baird et al. 2017; Juszkiewicz et al. 2022) are corals that were originally thought to be subtropical members of different, more cosmopolitan species. In Japan, the same pattern has been observed in the *Acropora hyacinthus* and *Goniopora lobata* species complexes (Nakabayashi et al. 2019; Yasuda et al. 2021; Fifer et al. 2022). Observations of morphologically distinct ‘tropical’ *Acropora* spp. having expanded into previously unrecorded high latitudes (30°S) (Baird et al. 2012) were deemed inconsequential, as subsequent benthic surveys concluded there was a lack of an overall increase in the abundance of tropical/cosmopolitan corals compared to the coral assemblage in 1992 (Mizerek et al. 2021). Since the growth, survival and recruitment rates of subtropical corals are different to their tropical counterparts (Cant et al. 2023; Chong et al. 2023), range expansion of tropical corals poleward would lead to differential population level responses to environmental disturbances, in turn affecting the persistence and ecological functioning of high-latitude reefs. It is therefore important to identify whether habitat forming species in ‘tropicalising’ biogeographic transition zones consist of poleward range expanding tropical species, or whether they are proliferating subtropical species, in addition to monitoring changes in coral and symbiont diversity and abundances.

*Pocillopora* is a genus of morphologically plastic scleractinian corals that are known to adjust their morphological structure based on environmental conditions (Paz-García et al. 2015; Soto et al. 2018), making them difficult to visually identify to species level based on morphological features alone. The flexible characteristics of *Pocillopora* corals suggest that they should be good candidates for both range expansion and subtropical proliferation at niche boundaries (Hoogenboom et al. 2008), and are therefore ideal to test between these two alternative hypotheses. However, genetic data are crucial for species delineation. Delineating haplotypes (clusters of genetic material co-located in the genome and are typically inherited together) based on known DNA markers (Gélin et al. 2017) gives intraspecific resolution for the visualisation of genealogical relationships. This allows the inference of biogeography and history of coral populations (Leigh and Bryant 2015). Species in *Pocillopora* span a wide spectrum of coral life-history traits: some are considered weedy and some competitive (Darling et al. 2012; Madin et al. 2016), while some are stress-tolerant (Haryanti et al. 2015; Fox et al. 2021). *Pocillopora* corals are able to recover rapidly after disturbances through high recruitment (Holbrook et al. 2018) and have been observed to dominate settlement panels at high-latitude reefs across different ocean basins (Nakamura et al. (2021)). The obligate coral–symbiont relationship might also play an important role in ensuring a good nutritional strategy for different environments in which *Pocillopora* corals are found. Photosynthetic Symbiodiniaceae dinoflagellates (hereafter symbionts) are essential for the growth and survival of corals. One coral colony may harbour several species of symbionts and may be able to adjust the abundances of their dominant symbionts based on the required thermal, light and oxygen sensitivity (Jones et al. 2008; Putnam et al. 2012; Wang et al. 2022), often with some *Durusdinium* spp. considered to be more beneficial under heightened heat stress. *Pocillopora* are brooders as well as broadcast-spawners (Ward 1992; Schmidt-Roach et al. 2012), suggesting a mixed transmission mode of symbionts in this genus (both horizontal and vertical) (Baird et al. 2009). This affords a higher degree of flexibility, which might be especially beneficial for coral persistence and survival under changing environmental conditions (Quigley et al. 2018, 2019; Baird et al. 2021). Indeed, *P. aliciae* (a subtropical endemic), previously thought to be *P. damicornis*, has proliferated at higher latitudes (33°S) off Sydney, Australia (O’Connell et al. 2023), and hosts endemic symbiont lineages (Schmidt-Roach et al. 2013a). Understanding *Pocillopora* range expansion and/or proliferation in other regions, such as the Kuroshio Current region in the northern Pacific Ocean, will therefore help promote better understanding of mechanisms of coral persistence at high latitudes under global climate change.

Despite being one of the most well studied genera of scleractinian corals, there is limited work linking the identities of *Pocillopora* host and symbionts (but see Wicks et al. (2010); Johnston et al. (2022); Millán-Márquez et al. (2024)) to how tropical–temperate populations respond to increasing environmental stress: either by the range expansion of tropical lineages, or by the proliferation of subtropical specialists. For any group of zooxanthellate corals, range expansion versus existing proliferation should show different genetic signatures in common coral genetic markers. The hypothesis of tropicalisation by recent range expansion predicts closely related haplotypes along the range expansion front, while range increasing via proliferation is expected to show a longer history of mutation accumulation and population structure (Slatkin and Hudson 1991). These patterns are likely to also be reflected in their dinoflagellate symbionts in co-phylogeny and host specificity (Johnston et al. 2022). Because of the symbionts’ critical role in facilitating the survival of coral hosts, we expected corals that inhabit high-latitude range limits to harbour distinctly different Symbiodiniaceae communities to those at lower latitudes. Here, we genotyped *Pocillopora* and its symbionts along a ~ 1500 km environmental gradient (24.3–33.5°N), from Iriomote Island in southern Japan, to Kushimoto, Wakayama on the Pacific coast of mainland Japan, following the Kuroshio Current. We aimed to (1) understand the identity of *Pocillopora* host and symbiont diversity along the environmental gradient and (2) test whether the genetic structure of *Pocillopora* and symbionts imply a range expansion poleward or a proliferation of subtropical lineages. Our results facilitate understanding and accurately projecting change in high-latitude coral assemblages under climate change.

## Material and methods

### Specimen collection

**Fig. 1:**
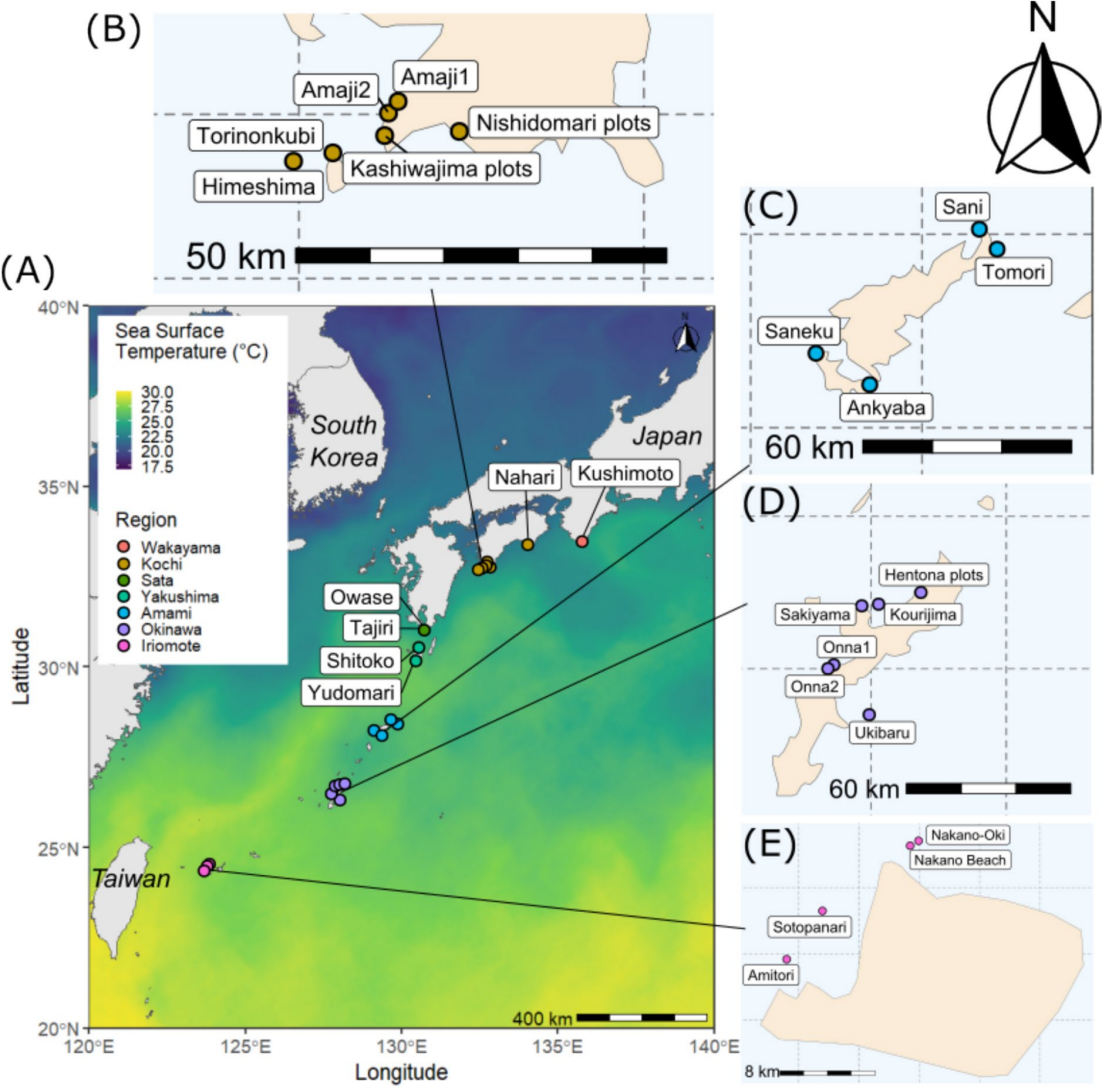
**A** The 26 sample sites superimposed on sea surface temperature (SST) data from 16 June 2023 (CoastWatch, Nasa/Jpl 2015). The Kuroshio Current can be traced northwards following the warmer temperatures (yellow) along the Ryukyu Islands and the coast of Japan. Country names are in italics. Sample site circles are coloured by region/major island with a total of seven regions from north to south: Wakayama, Kochi, Sata, Yakushima, Amami, Okinawa and Iriomote. Insets (B-E) show sampling sites that are too close to see clearly in (**A**). **B** Kochi, **C** Amami, **D** Okinawa and **E** Iriomote. For more information, see Supplementary Information

We collected coral tissues from 332 *Pocillopora* colonies from June to August 2023 from 26 sites: from the tropical coral reefs in Iriomote, Okinawa in Southern Japan to the subtropical communities of Kushimoto, Wakayama (Fig. 1, Table S1). At each site, following three 30 m transects at 5–10 m water depth, we aimed to sample at least five colonies per transect. Colonies sampled were at least 1 m away from each other in an attempt to avoid sampling clones. However, this was not always possible at sites where *Pocillopora* corals were not common. At those sites, fewer samples were obtained at similar water depths opportunistically. For each coral, we removed three to five verrucae from the branches using a bone cutter, avoiding growing tips. We also took two scaled photographs of each sampled colony: one showing the details of the corallites and one showing the gross morphology of the coral colony, using an Olympus Tough TG-6 camera (Olympus Corporation, Tokyo, Japan). We stored coral tissue samples in 99.1% ethanol until DNA extraction in October 2023.

### Characterising the Kuroshio Current environmental gradient

**Fig. 2:**
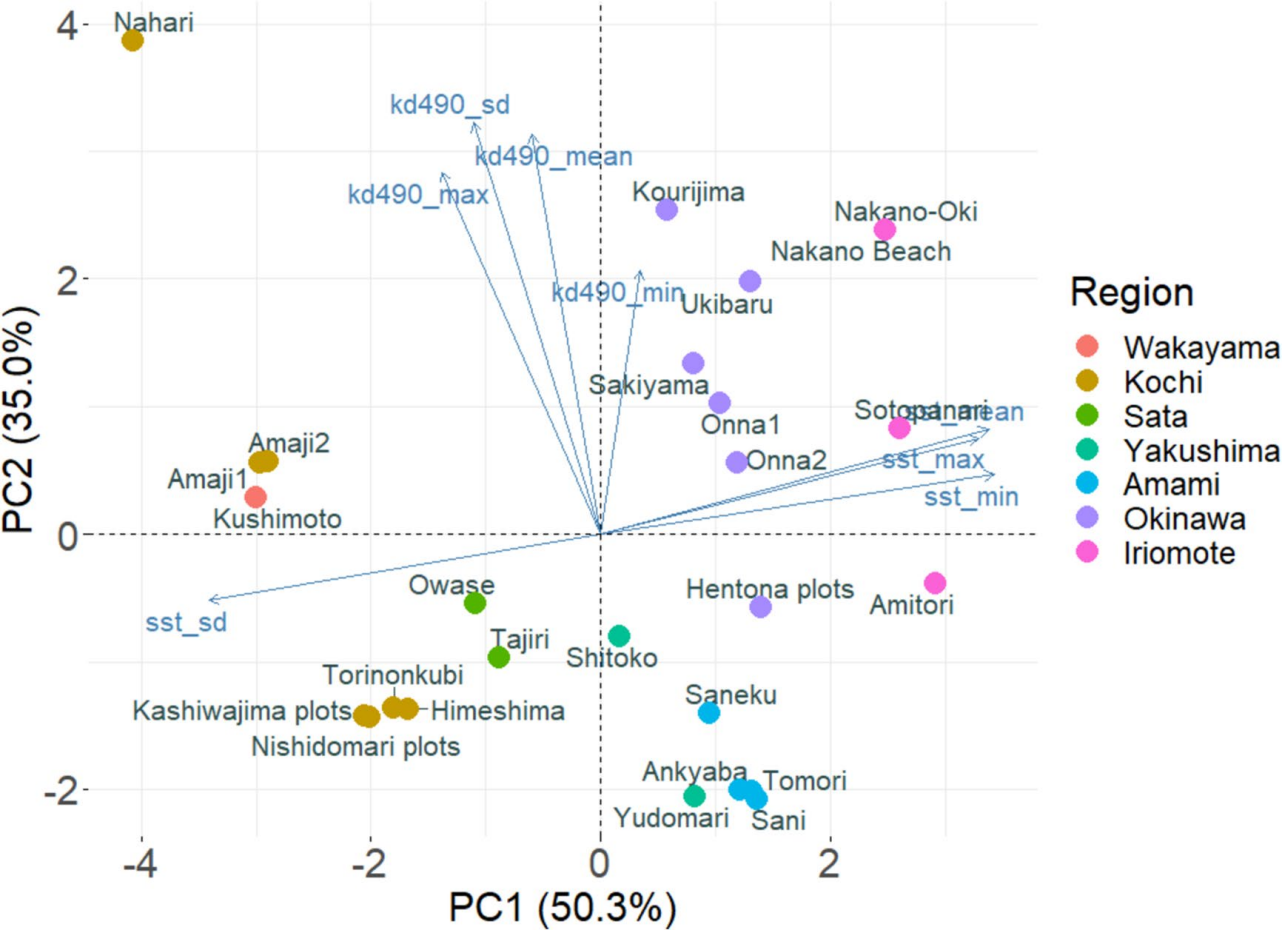
Principal component analysis of environmental conditions along the tropical-to-temperate transition in Japan. The eight environmental variables (blue arrows): sea surface temperature (sst) and turbidity (kd490) variables (minima, maxima, mean and standard deviation) on two principal component axes, which jointly explained 85.3% of the temperature-turbidity regime along this gradient. Site names (dots) coloured by geographic region as in Fig. 1. Positive PC1 scores represent hotter and less variable sites, while positive PC2 scores represent sites with more turbid waters that experienced high variability in turbidity

In Japan, the Kuroshio Current is a directional driver of the extension of tropical reef environments. We characterised the environmental gradient of all sites with a focus on seawater temperature and turbidity, due to their known influences on coral physiology which might drive coral distribution (Sully and van Woesik 2020; Abrego et al. 2021). We extracted monthly 1 km-resolution sea surface temperature (SST) (‘jplMURSST41mday’; Nasa/Jpl 2015) and 4 km-resolution kd490 (diffuse attenuation coefficient at 490 nm) as a proxy for turbidity (‘nesdisVHNSQkd490Monthly’; NOAA 2012), from CoastWatch using the packages ‘rerddap’ (Chamberlain 2024) and ‘rerddapXtracto’ (Mendelssohn 2024). We calculated the minima, maxima, means and standard deviations for the monthly SST and kd490 over the period January 2012–July 2023. We performed a principal component analysis (PCA) on these eight variables for dimension reduction (Fig. 2). The first axis (PC1) explained 50.3% of the observed variance, where positive scores reflect warmer sites that experienced less temperature fluctuations than colder, more variable sites. The second axis (PC2) explained 35.0% of the variance and captured a turbidity gradient. Positive PC2 scores indicated sites with murkier waters that also experienced higher variations in turbidity (Fig. 2). The PC1 and PC2 scores were then extracted for subsequently statistical analyses.

### DNA extraction, *Pocillopora* host sequencing and Symbiodiniaceae NGS

We extracted total genomic DNA from tissue using the DNeasy Blood and Tissue Kit (Qiagen, Hilden, Germany) following the manufacturer’s instructions. Using a NanoDrop ND-1000 spectrophotometer (Thermo Scientific), we quantified the quality and the concentrations of extractions and diluted each sample to a concentration of 1 ng/µl using UltraPureTM DNase/RNase free water (Invitrogen). We amplified the mitochondrial open reading frame (ORF) gene of the *Pocillopora* hosts with the FATP6.1 (forward) and RORF (reverse) markers, following the recommended polymerase chain reaction (PCR) cycles (Flot et al. 2008) (Table S2). DNA from 311 samples was successfully amplified and sent to FASMAC (Kanagawa) to be Sanger sequenced in both directions with 3730xl DNA Analyzer (Applied Biosystems, Thermofisher) in December 2023 and January 2024.

For the symbionts, we amplified the internal transcribed space two region of ribosomal DNA (ITS2 rDNA) from the same DNA extractions, with the primer pair SYM_VAR_5.8S2/SYM_VAR_REV, following the recommended PCR cycles (Hume et al. 2018) (Table S3), using TaKaRa *ExTaq*® Hot Start version (Takara Bio Inc., Shiga, Japan). After purification using Ampure XP beads (Beckman Coulter, Brea, CA, USA), we sequenced the amplified and indexed PCR products on the Illumina MiSeq platform at the National Institute of Advanced Industrial Science and Technology (AIST; Tsukuba, Japan), using a MiSeq Reagent Nano (v2-500 cycle) kit to generate 2 × 250 bp paired-end reads. We processed the obtained FASTQ files using the SymPortal analytical framework version 0.3.20 to determine Symbiodiniaceae genotypes (Hume et al. 2019). Briefly, individual corals may host Symbiodiniaceae from more than one genus, as well as different species and types from the same genus. Each Symbiodiniaceae cell contains multiple copies of the ITS2 gene, giving high intragenomic diversity. In SymPortal, recurring sets of ITS2 sequences are called defining intragenomic variants (DIVs) and their abundances are catalogued. The relative abundance and combinations of DIVs then contribute to the definition of unique ITS2 profiles, which can be considered symbiont ‘taxa’ (Hume et al. 2019). In SymPortal, sequence quality control is performed using mothur, the BLAST + suite and minimum entropy decomposition (MED).

### Haplotype network and Phylogenetic tree of *Pocillopora* ORF haplotypes

We trimmed and aligned 311 forward and reverse sequences of *Pocillopora* using MUSCLE on Geneious 11.0.5 (https://www.geneious.com), inspecting single nucleotide changes manually to verify sequence base calling. We drew a haplotype network with median joining network (Bandelt et al. 1999) using PopART (Leigh and Bryant 2015). Using the Geneious Tree Builder, we built a phylogenetic tree (HKY Model, neighbour-joining tree build method, with bootstrap resampling (random seed) and 100 replicates to create consensus trees), to examine the relationship amongst mitochondrial ORFs found in our study region (Fig. S1).

### Testing the effect of the environmental gradient on coral and symbiont diversity

To understand the effect of environmental conditions on the number of haplotypes present at a given site, we ran an ordinal logistical regression using the function polr() from the ‘MASS’ package (Venables and Ripley 2002). We used PC1 and PC2 scores as predictor variables, and the number of *Pocillopora* haplotypes as a categorical response variable to predict the probability of finding a given number of haplotypes at a site. Using chisq.test() and the option to simulate p-values, we checked the goodness of fit and generated a Nagelkerke pseudo R2 value using PseudoR2() from the package ‘DescTools’ (Signorell 2024). We also calculated p-values and confidence intervals for each parameter estimate (Table S5). Holding PC2 scores at the mean, we predicted the probabilities of the number of haplotypes found over the range of PC1 scores. 

Similarly, to test the effect of environmental conditions (PC1 and PC2 scores) on the diversity and abundance of symbiont communities (DIVs) at each site, we ran a distance-based redundancy analysis (dbRDA) using capscale()with Bray–Curtis distance from the package ‘vegan’ (Oksanen et al. 2022). The model significance was tested using anova(). 

## Results

### Haplotype diversity and changes along the environmental gradient

**Fig. 3:**
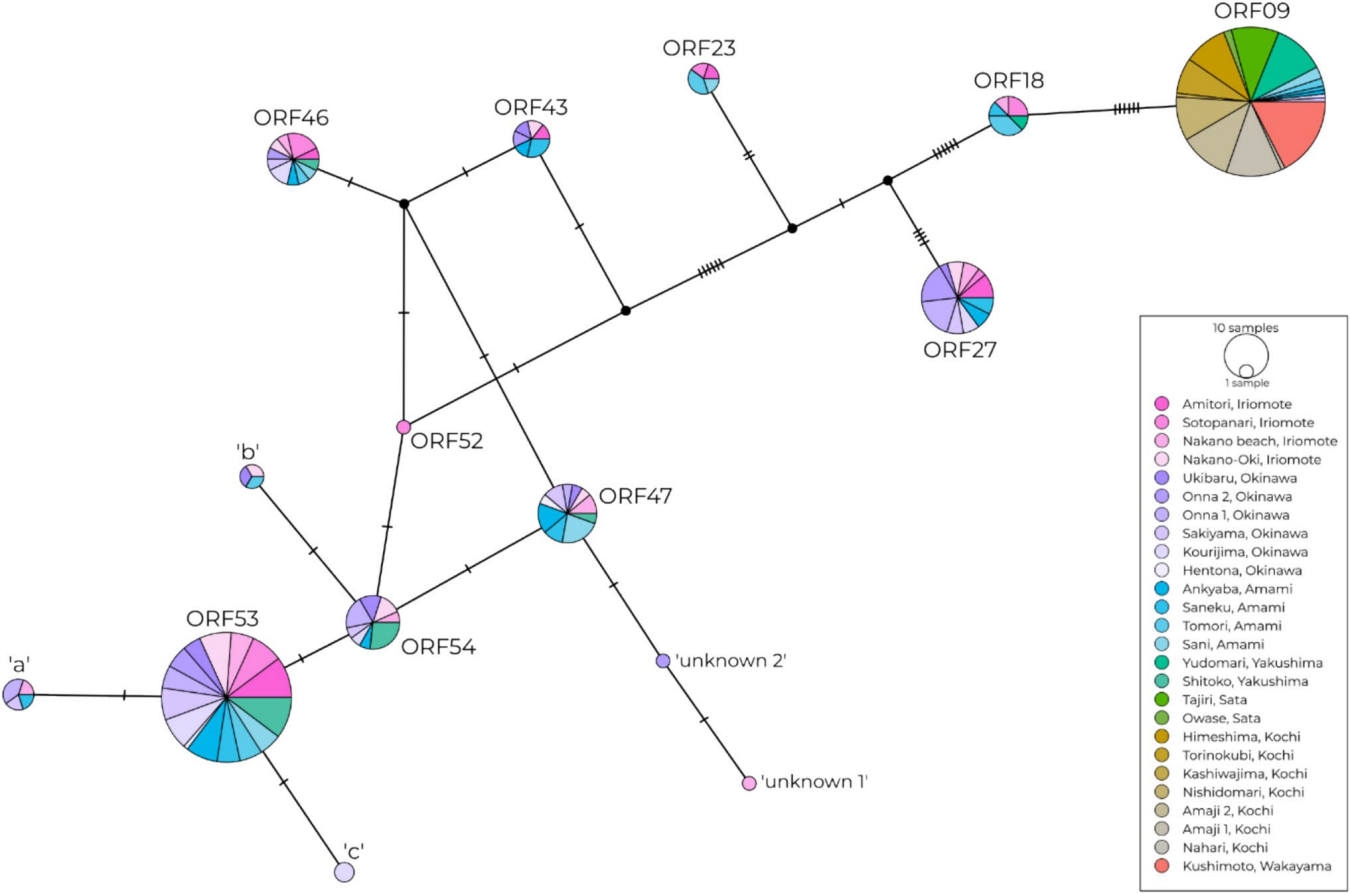
Haplotype network (Median Joining Network: MJN) of *Pocillopora* ORF haplotypes (n = 311). Vertical bars (hatches) represent the number of base pair differences between haplotypes. Black circles are unsampled ancestors inferred by the MJN algorithm. The size of each pie indicates the number of samples found to have that haplotype. The colours correspond to the seven regions as in Fig. 1: Iriomote, Okinawa, Amami, Yakushima, Sata, Kochi and Wakayama

**Fig. 4:**
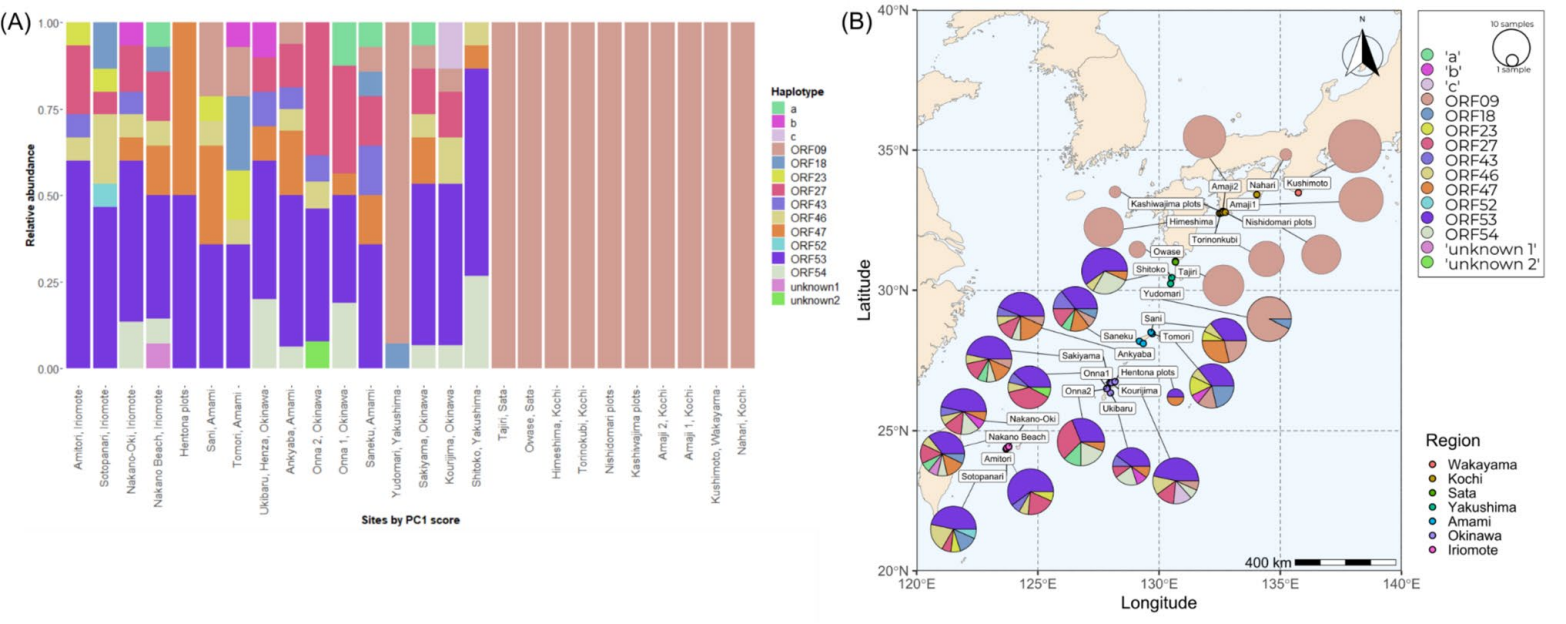
**A** The relative abundances of *Pocillopora* mtORF haplotype at each site, ordered by PC1 score (left to right: high to low PC1; high PC1 means higher SST). Haplotype nomenclature follows (Gélin et al. 2017). New sequences identified in this study ‘a’, ‘b’ and ‘c’ were found in more than one coral sample, while ‘unknown1’ and ‘unknown2’ are sequences found only in one sample (n = 311). **B** The haplotypes and abundances are displayed by sampling location on a map, with sample sites coloured by region as in Fig. 1. Haplotypes are coloured as in (**A**)

We found a total of 15 *Pocillopora* haplotypes, of which 10 have previously been recorded (Gélin et al. 2017) (Fig. 3). ORF09 and ORF53 were the two haplotypes found with the highest frequencies, while ORF53 was found exclusively in the southern islands (Iriomote, Okinawa, Amami and Yakushima). These two haplotypes were separated by 22 base pair differences, which corresponded to a separation of over 1.16 million years (average time for a mitochondrial genome mutation to occur is approximately 53,000 years in *Pocillopora* corals; (Palumbi et al. 2023)). The southern island sites had high haplotype diversity (n = 15), while mainland Japanese sites (Sata, Kochi and Wakayama) only had one haplotype (ORF09; Fig. 4). On Yakushima Island (the island closest to mainland Japan), the site Yudomari also hosted mostly ORF09, with one sample of ORF18. According to Gélin et al. (2017)‘s primary species hypothesis (PSH) species delimitation method, the 10 known haplotypes constituted seven ‘PSH’s (species), with a range of possible morphological classifications (Table S4). Specifically, ORF09 was considered to be *damicornis*-like, while ORF53 had at least five different morphotypes including *verrucosa*-like morphotypes.

**Fig. 5:**
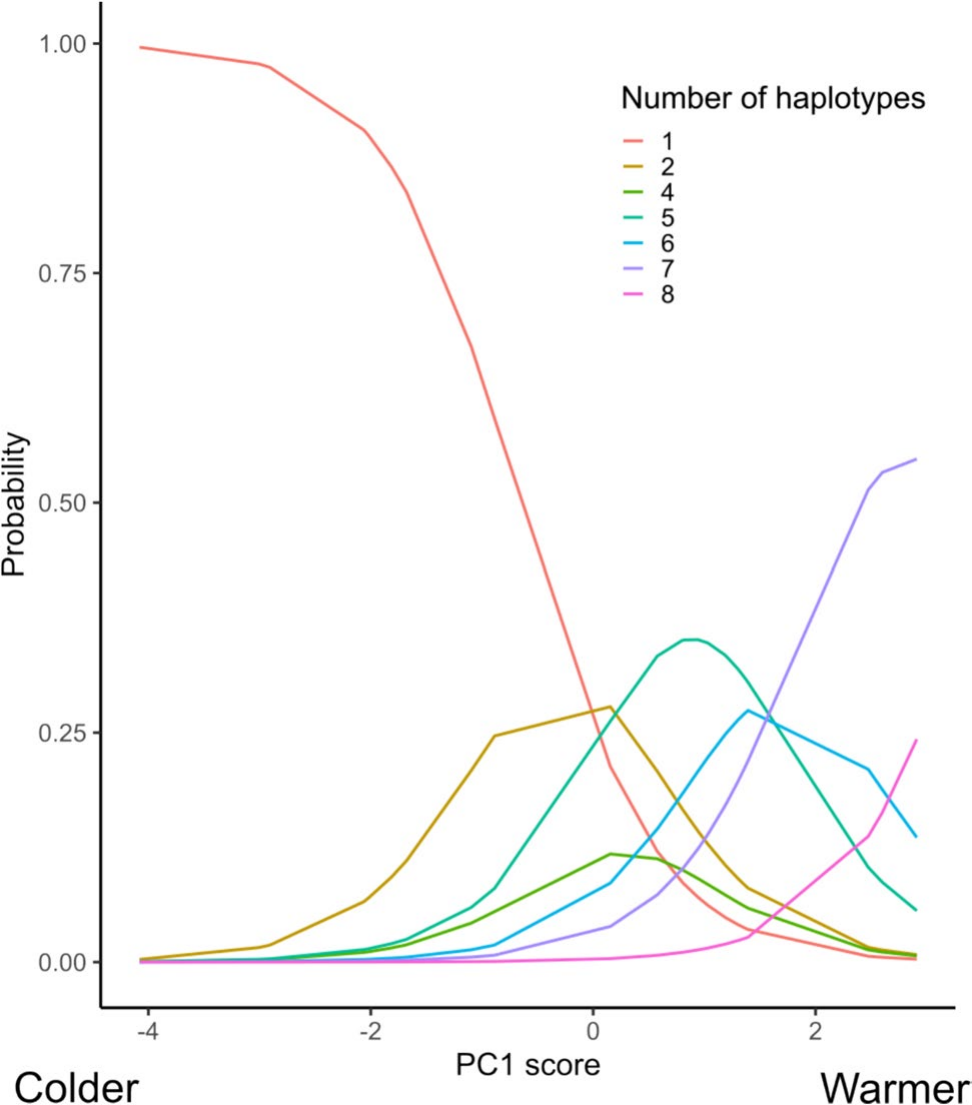
Predicted probability of a given number of *Pocillopora* haplotypes at a site along the temperature gradient: more positive PC1 scores represent warmer water temperatures and less variability in water temperatures. PC2 score was held at its mean value for the regression. Number of haplotypes are 1–2, 4–8, because there were no sites with three haplotypes in our dataset

The number of *Pocillopora* haplotypes decreased with increasingly colder and more variable environments (negative PC1 scores) (Fig. 5). Ordinal logistic regression showed that PC1 scores, reflecting a sea surface temperature gradient, was a significant predictor of the number of haplotypes, while PC2 scores (capturing a turbidity gradient) was not significant (Nagelkerke pseudo R2 = 0.726, Table S5). Most changes in the number of haplotypes were predicted to occur at sites with PC1 values around 0–2, (*i.e.* the islands of Okinawa, Amami and Yakushima), where the probability of finding only one haplotype fell dramatically, and finding two to six haplotypes peaks. At the warmest sites (PC1 scores of > 2; Iriomote island), there was a higher probability of finding seven or more haplotypes.

### Symbiont defining intragenomic variants (DIVs) and ITS2 type profile along the Kuroshio

**Fig. 6:**
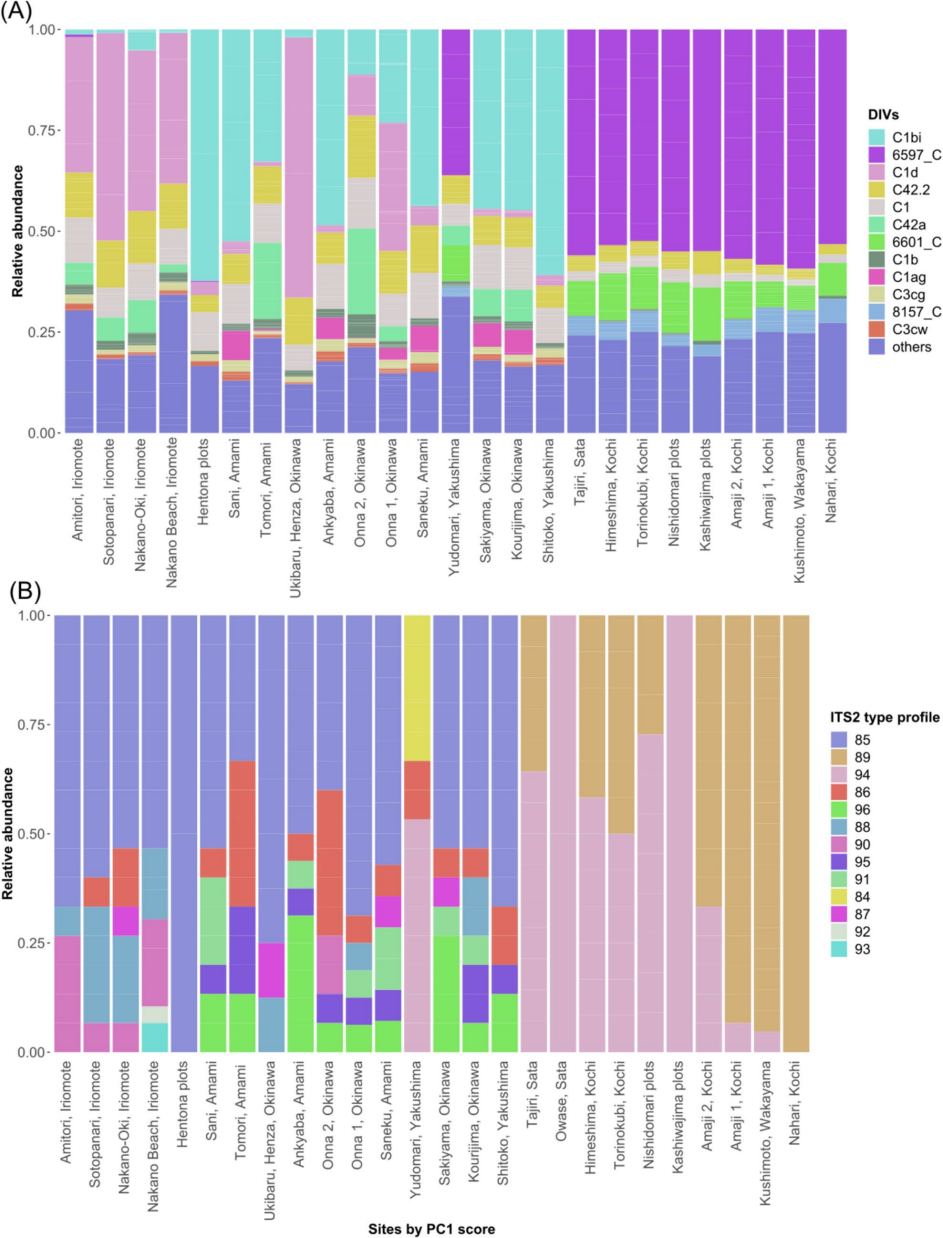
**A** The relative abundances of the top 12 most abundant Symbiodiniaceae defining intragenomic variants (DIVs), ordered by PC1 score (left to right: high to low PC1; high PC1 means higher SST). The remainder DIVs were all classified under the group ‘others’. The most abundant DIVs were all of the genus *Cladocopium*. A number followed by _C e.g. 6598_C are unnamed species of *Cladocopium.* **B** The relative abundances of the dominant ITS2 type profiles, ordered by PC1 score (left to right: high to low PC1; high PC1 means higher SST). For the DIVs that make up each ITS2 type profile, see Table S6. All ITS2 type profiles are dominated by *Cladocopium*, except for 93, which was *Durusdinium glynii* dominant

**Fig. 7:**
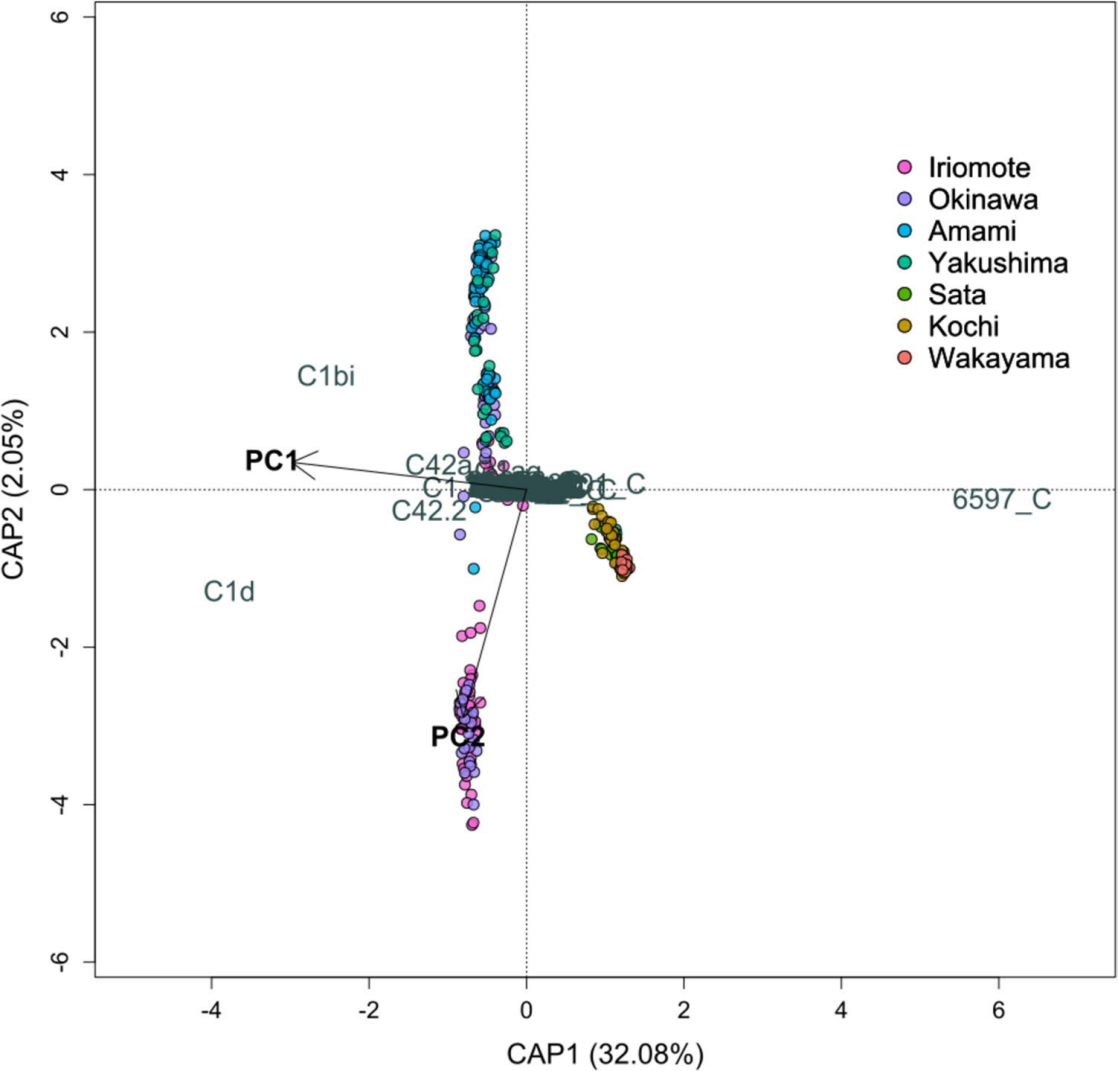
Distance-based redundancy analysis (dbRDA) (Bray–Curtis distance, capscale()) triplot showing the symbiont DIVs (response variables) against PC1 and PC2 scores (predictor variables). The circles are coral samples coloured by the geographic region that they were collected from along the tropical-to-temperate transition in Japan

Symbiont DIVs were different between the southern islands and mainland Japan. While the lower latitude sites had more *Cladocopium* C1d and C1bi, mainland reefs mainly hosted unnamed *Cladocopium* species with the unique identifiers 6597_C and 6601_C (Fig. 6A). The corresponding ITS2 type profiles also mirrored the pattern observed for DIVs (Fig. 6B). Only two ITS2 type profiles were found on mainland Japan with UIDs 89 and 94 and were both *Cladocopium* 6597_C and 6601_C dominant; while 11 other ITS2 type profiles were found on the southern islands, mostly consisting of combinations of DIVs *Cladocopium* C1b, C1d, C42a, C1 (for the dominant DIVs of each ITS2 type profile, see Table S6). *Durusdinium glynnii*, a heat adapted species (93), was found at Nakano Beach on Iriomote. Yudomari (Yakushima) corals hosted a unique ITS2 type profile (84) made up of a majority sequence of an unnamed *Cladocopium* DIV 25378_C and C1c. dbRDA showed that both PC1 and PC2 (SST and turbidity) were significant predictors for the symbiont DIV community composition with an adjusted R^2^ = 0.354 (Fig. 7). Higher SSTs (positive PC1) increased the proportion of *Cladocopium* C1d, C1bi, C1, C42a and C42.2, but limited *Cladocopium* 6597_C, while the remaining 932 DIVS were clustered near the origin with a high degree of overlap (See supplementary data for all DIVs).

## Discussion

Tropicalisation and the resultant reorganisation of benthic communities could transform ecological processes and interactions at tropical–temperate biogeographic transition zones. While benthic changes have been recorded along western boundary currents at tropicalisation hotspots (Vergés et al. 2014, 2016; Kumagai et al. 2018), the identity of scleractinian corals that have the potential to expand and proliferate has not often been clearly ascertained, leading to claims that tropical corals could find refugia at higher latitudes (de Oliveira Soares 2020). In this study, we established that *Pocillopora* corals and their symbionts along the Kuroshio Current are different in the Southern Ryukyu Islands compared those at mainland Japan sites, which has only one *Pocillopora* haplotype and a distinct symbiont profile. Our results are consistent with the idea that temperate coral communities may largely consist of subtropical endemics and specialists, rather than representing extensions of tropical species that are expanding their range (*i.e.* tropicalisation) due to anthropogenic climate change (Keshavmurthy et al. 2023). Indeed, *Pocillopora* corals have been recorded on mainland Japan since 1930s (Yabe and Sugiyama 1935). As climate change leads to ocean warming, it is unclear how subtropical endemics and specialists will fare, due to their lower thermal threshold for high temperatures and thus increased susceptibility to bleaching (Kim et al. 2019). We find no evidence that other haplotypes of *Pocillopora* can tolerate the colder and more variable conditions in subtropical/temperate regions along the Kuroshio Current off mainland Japan, adding nuance to the general marine tropicalisation discussion.

The subtropical *Pocillopora* haplotype ORF09 was the only haplotype found in the colder waters of mainland Japan, although they were also present in much lower relative abundances at the southern island sites of this study. We hypothesise that the warmer and milder sea conditions from Yakushima Island southwards benefit more competitive *Pocillopora* haplotypes and their associated symbionts, allowing them to outcompete ORF09, which might be more cold stress-tolerant and is thus the only haplotype that persists in high-latitude marginal reefs. Our evidence also points towards a mismatch between the location of the ‘Tokara Gap’ (a biogeographic boundary) and coral diversity in this region, and we join the call for more careful examination of biodiversity in biogeographic transition zones (Komaki 2021). Our haplotype network did not form a single ‘starburst' as would be expected if a single population had recently evolved and rapidly expanded along an increasingly suitable environmental gradient (Slatkin and Hudson 1991). ORF09 is genetically different to its closest relative ORF18 by six base pairs, suggesting that the populations of these haplotypes have been separated for approximately 318,000 years (53,000 years/mitochondrial mutation in corals, at 0.1% per million years (Palumbi et al. 2023)), while haplotypes found in the southern islands are more closely related, many with only one base pair difference between them. ORF09 is also host to subtropical unnamed *Cladocopium* symbiont DIVs 6597_C and 6601_C, whose dominance in the DIV composition gave unique subtropical combinations of ITS2 types. This finding is in broad agreement with Lien et al. (2013, Fig. 5), who had previously found distinctly different symbiont ITS2 types in *P. damicornis* colonies sampled from Shirahama on mainland Japan, and the southern islands of Amami and Sesoko. Symbiodiniaceae differences along the Kuroshio current have also been observed in other zooxanthellate anthozoans (Reimer et al. 2006).

As one would expect to find many closely related haplotypes on a range expansion front, it is possible that tropicalisation of *Pocillopora* is happening but has not yet reached the subtropical/temperate reefs of mainland Japan. It is theoretically possible that the southern islands (e.g. Okinawa and Amami) hosted fewer *Pocillopora* haplotypes until recently, and might host different symbiont communities. If historical samples are available, it will be worthwhile to extract, amplify and sequence ‘ancient’ DNA to understand the effect of anthropogenic environmental change (Baker et al. 2013). Continual monitoring in the entire Kuroshio region would improve our understanding of the mechanisms of coral population persistence along biogeographic transition zones. From our results, Yakushima Island might represent the best location to monitor for tropical haplotype expansion. Of the two Yakushima sites visited, Yudomari hosted predominantly the subtropical haplotype (ORF09) and a unique symbiont ITS2 type profile (84) dominated by 25378_C and C1c, while Shitoko hosted no ORF09, but other haplotypes that were found in the southern island groups, with symbiont ITS2 profiles that resemble those from Okinawa. Reefs on Yakushima Island thus may be one of the potential stepping stones (Saura et al. 2014) for poleward expansion of the ‘tropical’ *Pocillopora* haplotypes and associated symbionts under climate change.

Although we did not explicitly test the physiological performance of the different haplotypes and their symbionts (e.g. Edmunds et al. 2024), environmental differences clearly predict haplotype diversity and distribution (e.g. Figs. 5, and 7). The subtropical symbiont DIVs recorded in this study demonstrate that there are specific coral–symbiont types and communities that are better suited to colder and more turbid temperate sites. In addition to heat stress experiments that are more commonly employed to understand coral–symbiont physiology, low temperature, light availability and salinity stress experiments (e.g. Kerswell and Jones 2003; Jones et al. 2020) with different symbiont communities could be a worthy endeavour in understanding the physiology of corals in marginal reefs. For instance, it would be useful to establish the survival rates of tropical-subtropical symbionts in the water column to see if they remain viable under different environmental conditions, to be taken up by prospective coral hosts. It is also pertinent to understand the reproductive and survival strategies of these different *Pocillopora* haplotypes on the range expansion front, to predict if beneficial symbionts can be taken up from the environment (horizontal transmission) as well as inherited from the mother (vertical transmission). Although we show that there is host specificity between *Pocillopora* haplotype and associated symbionts, poleward range expansion of tropical haplotypes might be promoted if there can be an active uptake of subtropical symbiont communities from the environment. Understanding these physiological differences could improve our ability to project population performances under climate change (Cant et al. 2022).

To our knowledge, Wakayama is the most northern location that *Pocillopora* has been observed in Japan. But based on environmental parameters, coastal locations are likely to experience more marginal (colder and more turbid) conditions. For example, in Nahari, Kochi, coral communities experienced heavy mortality in a 2018 cold bleaching event, when the Kuroshio Current meandered away from the coast (Leriorato and Nakamura 2019). Climate change is expected to affect the strength of western boundary currents (WBCs), and the Kuroshio Current is projected to weaken (Sen Gupta et al. 2021). It is therefore possible that extremely cold winter conditions could appear in sites on mainland Japan in the future, leading to heavy coral mortality that could take years to recover from. This further hampers the likelihood of tropicalisation by range expansion of tropical lineages at poleward limits. Adding genetic data from more tropical sites, such as in Taiwan (Hsiao et al. in prep) and the Philippines (Torres and Ravago-Gotanco 2018) could improve our understanding of the level of connectivity, diversity and distribution of *Pocillopora* along the Kuroshio environmental gradient. Such research would help identify regions of high genetic diversity or potential stepping stones of any poleward range expansion. In other parts of the world, WBCs such as the Brazil Current and the East Australian Extension are predicted to strengthen (Sen Gupta et al. 2021). The increased transport of warmer waters poleward could encourage the range expansion of coral taxa in those regions. This process could have unknown or potentially negative effects on native subtropical and temperate endemics (Martello et al. 2024) by augmenting benthic composition with knock-on effects on ecosystem functioning of high-latitude reefs.

Climate change and ocean warming will continue to affect ecological communities, including via tropicalisation, where tropical taxa expand their range poleward for more favourable environmental conditions. Although frequently observed in mobile taxa such as marine fish (Vergés et al. 2016; Miller et al. 2023; O’Connell et al. 2023), there is mixed evidence for tropicalisation by range expansion in zooxanthellate reef corals, likely due to cold extremes during winter months in poleward locations, making those sites unviable even for subtropical corals, let alone tropical lineages. Recent observations of coral expansion in the subtropics could be due to the scientific community’s increased awareness of (subtropical endemic) corals at high latitudes, and/or increasingly favourable conditions for subtropical coral proliferation due to warming. Our study of *Pocillopora* spp. and their symbionts in the Kuroshio region highlights the taxonomic distinctness of subtropical endemics and their long evolutionary history adapting to local environments (Thomas et al. 2017). It is unclear whether high-latitude regions can support tropical corals and act as climate refugia for tropical coral reef biodiversity, but we show that the evolutionary distinctness of subtropical communities (Budd and Pandolfi 2010) underpins fundamental differences to their tropical counterparts.

## Supplementary Information

Below is the link to the electronic supplementary material.

Supplementary file1 (DOCX 99 kb)

## Data Availability

DNA sequences generated and analysed during the current study will be made available on GenBank, https://www.ncbi.nlm.nih.gov/. All other datasets generated in this study are available from the corresponding author on reasonable request.
