# Supplementary material for: Subtropical specialists dominate a coral range expansion front
Source: Coral Reefs. 2024 Dec 16;45(1):5–19. doi: 10.1007/s00338-024-02601-w (PMC12916933; doi:10.1007/s00338-024-02601-w)
Supplement: Supplementary file 1 — Supplementary file1 (DOCX 99 kb) [file 338_2024_2601_MOESM1_ESM.docx]

## Supporting information

Table S1. The 26 sampling site names, their geographic regions, their GPS co-ordinates in decimal degrees, the sampling date (yyyy/mm/dd) and the number of coral individuals sampled per site. Asterisks (*) indicate the sites that were sampled opportunistically.

| Site name | Region | Latitude | Longitude | Sampling date (yyyy/mm/dd) | Coral individuals sampled |
| --- | --- | --- | --- | --- | --- |
| Nakano Beach | Iriomote | 24.43152 | 123.7924 | 2023/06/09 | 15 |
| Amitori | Iriomote | 24.34577 | 123.69 | 2023/06/10 | 15 |
| Sotopanari | Iriomote | 24.38228 | 123.7196 | 2023/06/10 | 15 |
| Nakano-Oki | Iriomote | 24.43522 | 123.7993 | 2023/06/11 | 16 |
| Ukibaru | Okinawa | 26.34987 | 127.9936 | 2023/06/08 | 10 |
| Sakiyama | Okinawa | 26.70673 | 127.9662 | 2023/06/13 | 15 |
| Kourijima | Okinawa | 26.71213 | 128.0281 | 2023/06/13 | 16 |
| Onna1 | Okinawa | 26.51351 | 127.8627 | 2023/06/15 | 16 |
| Onna2 | Okinawa | 26.50093 | 127.8409 | 2023/06/15 | 15 |
| Hentona plots* | Okinawa | 26.75058 | 128.1848 | 2023/08/12 | 2 |
| Tomori | Amami | 28.46129 | 129.7186 | 2023/07/08 | 15 |
| Saneku | Amami | 28.19227 | 129.1905 | 2023/07/09 | 15 |
| Ankyaba | Amami | 28.11133 | 129.3473 | 2023/07/09 | 16 |
| Sani | Amami | 28.51297 | 129.667 | 2023/07/10 | 15 |
| Shitoko | Yakushima | 30.44893 | 130.5214 | 2023/07/11 | 16 |
| Yudomari | Yakushima | 30.23356 | 130.4736 | 2023/07/12 | 15 |
| Owase | Sata | 31.03699 | 130.6757 | 2023/07/14 | 2 |
| Tajiri | Sata | 31.00671 | 130.6765 | 2023/07/14 | 14 |
| Amaji2 | Kochi | 32.80153 | 132.63 | 2023/07/18 | 15 |
| Amaji1 | Kochi | 32.81577 | 132.6438 | 2023/07/18 | 15 |
| Himeshima | Kochi | 32.74289 | 132.4923 | 2023/07/19 | 12 |
| Torinokubi | Kochi | 32.75311 | 132.5493 | 2023/07/19 | 12 |
| Kashiwajima plots* | Kochi | 32.77418 | 132.6244 | 2023/08/24 | 1 |
| Nishidomari plots* | Kochi | 32.77917 | 132.7327 | 2023/08/22 | 11 |
| Nahari | Kochi | 33.41154 | 134.0309 | 2023/07/21 | 1 |
| Kushimoto | Wakayama | 33.47938 | 135.7461 | 2023/07/24 | 21 |

Table S2. Pocillopora PCR reagents and volumes used per sample, and PCR conditions following (Flot et al., 2008).

| Reagent | Volume (µl) | Stages | Temperature (°C) | Time (minute: second) |
| --- | --- | --- | --- | --- |
| HotStarTaq master mix | 10 | 1 | 94 | 1:00 |
| FATP6.1 | 1 | 2 (40 cycles) | 94 | 0:30 |
| RORF | 1 |  | 53 | 0:30 |
| CoralLoad | 2 |  | 72 | 1:15 |
| H_2_O | 5 | 3 | 72 | 5:00 |
| Genomic DNA | 1 |  |  |  |
| *Total volume* | *20* |  |  |  |

Table S3. Symbiodiniaceae PCR reagents (* indicates items that are part of the TaKaRa Ex Taq® Hot Start kit) and volumes used per sample and PCR conditions used, adapted from Hume et al. (2018).

| Reagent | Volume (µl) | Stages | Temperature (°C) | Time (minute: second) |
| --- | --- | --- | --- | --- |
| 10x *ExTaq* buffer* | 2 | 1 | 98 | 2:00 |
| dNTP mixture* | 1.6 | 2 (35 cycles) | 98 | 0:10 |
| SYM_VAR_5.8S2 | 1.2 |  | 56 | 0:30 |
| SYM_VAR_REV | 1.2 |  | 72 | 0:30 |
| *ExTaq* HS* | 0.2 | 3 | 72 | 7:00 |
| H_2_O | 12.8 |  |  |  |
| Genomic DNA | 1 |  |  |  |
| *Total volume* | *20* |  |  |  |


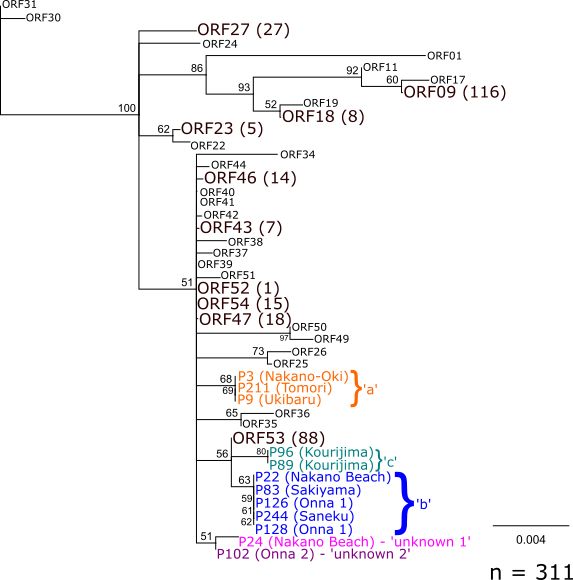


Figure S1. Phylogenetic tree showing the relationship between selected known haplotypes (Gélin et al., 2017). Large, dark brown fonts indicate haplotypes that were found in this work, followed by the number of corals in brackets found to be in that haplotype. Coloured fonts mark previously unrecorded sequences/haplotypes found in this study, showing the sample number and the location. ‘a’ was found in three samples, ‘b’ in five, ‘c’ in two; while ‘unknown 1’ and ‘unknown 2’ are singletons.

Table S4. ORF haplotypes found in this study and the associated PSH, SSH and possible morphotypes according to Table 3 of Gélin et al. (2017).

| ORF | PSH | SSH | Possible morphotypes according to SSH |
| --- | --- | --- | --- |
| 09 | 4 | 4 | damicornis |
| 18 | 5 | 5a-d | acuta, brevicornis, eydouxi, verrucosa |
| 23 | 6 | 6 | damicornis, verrucosa, elegans, meandrina |
| 27 | 9 | 9a-c | eydouxi, meandrina, woodjonesi, verrucosa, molokensis, damicornis, zelli |
| 43 | 13 | 13a | verrucosa, damicornis, meandrina, eydouxi, molokensis, kelleheri, zelli |
| 46 | 13 | 13b | verrucosa, damicornis, eydouxi, meandrina, molokensis, kelleheri |
| 47 | 13 | 13c | verrucosa, kelleheri, damicornis, meandrina |
| 52 | 15 | 15 | damicornis |
| 53 | 16 | 16 | verrucosa, damicornis, kelleheri, lingulata, meandrina |
| 54 |  |  |  |

Table S5. Parameter estimates, p value and confidence interval of the ordinal logistical regression (model <- polr(rank ~ PC1+PC2)), where PC1 and PC2 scores are predictor variables, and ‘rank’ (the different number of haplotypes) is the response variable. All values are rounded to three significant figures.

|  | Value | Std. Error | t value | p value | CI (2.5%, 97.5%) |
| --- | --- | --- | --- | --- | --- |
| PC1 | 1.61 | 0.440 | 3.66 | < 0.001 | 0.890, 2.65 |
| PC2 | 0.409 | 0.288 | 1.42 | 0.156 | -0.150, 0.997 |
| 1\|2 | -1.06 | 0.804 | -1.32 | 0.188 |  |
| 2\|4 | 0.213 | 0.711 | 0.300 | 0.764 |  |
| 4\|5 | 0.692 | 0.706 | 0.980 | 0.327 |  |
| 5\|6 | 2.162 | 0.753 | 2.872 | 0.004 |  |
| 6\|7 | 3.362 | 0.862 | 3.899 | < 0.001 |  |
| 7\|8 | 5.826 | 1.438 | 4.052 | < 0.001 |  |

Table S6. Defining Intragenomic Variants (DIVs) that make up symbiont ITS2 type profiles, ordered from high to low abundance. The total number of samples included are 325. Note that sample P26 fed into both type 88 and 92.

| **ITS2 type profile UID** | **85** | **89** | **94** | **86** | **96** | **88** | **90** | **95** | **91** | **84** | **87** | **92** | **93** |
| --- | --- | --- | --- | --- | --- | --- | --- | --- | --- | --- | --- | --- | --- |
| Clade | C | C | C | C | C | C | C | C | C | C | C | C | D |
| ITS2 type profile | C1bi/C1d-C1-C42.2-C3cg-C3cw-C1b | 6597-6601-8157-8156-C42.2 | 6597-6601-C42.2-8157-C1-8181 | C42a-C42.2-C1-C1b-C1au | C1bi-C1-10319-C42.2-10334 | C1d/C1-C42.2-C3cg-C1b | C42u/C42a-C1-C42.2 | C42a-C1-C42.2-C42b-C1b-8149 | C1ag/C42.2-C1-C3cg | 25378_C/C1c-C42.2-C1-4390_C-6682_C | C42a-C1-C42.2-C1b-8236 | C1bb | D1 |
| Majority ITS2 sequence | C1bi/C1d | noName | noName | C42a | C1bi | C1d/C1 | C42u/C42a | C42a | C1ag/C42.2 | noName/C1c | C42a | C1bb | D1 |
| Associated species | None | None | None | None | None | S. goreaui | None | None | None | None | None | None | S. glynnii |
| ITS2 profile abundance local | 115 | 64 | 48 | 23 | 19 | 15 | 11 | 11 | 9 | 5 | 4 | 1 | 1 |

References

Flot, J.-F., Magalon, H., Cruaud, C., Couloux, A., & Tillier, S. (2008). Patterns of genetic structure among Hawaiian corals of the genus Pocillopora yield clusters of individuals that are compatible with morphology. *Comptes Rendus. Biologies*, *331*(3), 239-247. <https://doi.org/10.1016/j.crvi.2007.12.003>

Gélin, P., Postaire, B., Fauvelot, C., & Magalon, H. (2017). Reevaluating species number, distribution and endemism of the coral genus Pocillopora Lamarck, 1816 using species delimitation methods and microsatellites. *Mol Phylogenet Evol*, *109*, 430-446. <https://doi.org/10.1016/j.ympev.2017.01.018>

Hume, B., Ziegler, M., Poulain, J., Pochon, X., Romac, S., Boissin, E., de Vargas, C., Planes, S., Wincker, P., & Voolstra, C. (2018). An improved primer set and amplification protocol with increased specificity and sensitivity targeting the Symbiodinium ITS2 region. *PeerJ 6*, *e4816*. <https://doi.org/https://doi.org/10.7717/peerj.4816>
